# The next step: detailed assessment of an adult glaucoma patient

**Published:** 2012

**Authors:** Heiko Philippin, Peter Shah, Matthew Burton

**Affiliations:** Head of postgraduate training and glaucoma specialist: Kilimanjaro Christian Medical Centre, Moshi, Tanzania. Email: philippin@gmx.de; Ophthalmologist and glaucoma specialist, NHS Foundation Trust Birmingham, UK, and Centre for Health and Social Care Improvement, University of Wolverhampton, School of Health and Wellbeing, UK. Email: Peter.shah@uhb.nhs.uk; Senior Lecturer, International Centre for Eye Health, London School of Hygiene and Tropical Medicine, London, UK.

**Figure:**
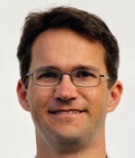
Heiko Philippin

**Figure:**
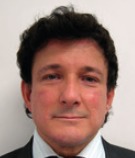
Peter Shah

**Figure:**
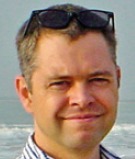
Matthew Burton

## Introduction

The glaucomas are a group of progressive optic neuropathies associated with characteristic structural changes at the optic nerve head (cupping) and corresponding visual field defects. The main modifiable risk factor for glaucomatous optic neuropathy is increased intraocular pressure (IOP). The aims of assessment are:

- accurate diagnosis
- identification of the cause of increased IOP, if applicable
- quantification of the level of glaucoma damage and functional impairment.

Although there are many possible causes of glaucoma (e.g. trauma, inflammation, previous surgery, or an inherited tendency), it is usually possible to identify the mechanism of elevated IOP by careful history taking, anterior segment examination, and optic disc assessment using a slit lamp.

After assessment, the clinician can group the glaucomatous optic neuropathies into three main categories: primary open-angle glaucoma (POAG), primary angle-closure glaucoma (ACG), and secondary glaucomas (including pseudo-exfoliation, pigmentary, uveitic, lens-induced, neovascular, steroid-induced and traumatic).

As a group, the glaucomas are chronic, life-long diseases, and it is therefore essential to collect and record clinical data accurately so that patients with progressive disease or who develop other ocular pathology can be identified at an early stage.

## History

Taking a careful history helps in two ways:

1. Identification of risk factors for glaucoma and glaucoma progression.
2. Identification of medical and social factors critical to optimum glaucoma management.

### Risk factors for glaucoma

- High IOP
- Age
- Ethnicity (African: POAG, Asian: ACG)
- Positive family history of glaucoma
- Refractive status (myopia and hypermetropia)
- Previous ocular trauma
- Previous intraocular inflammation
- Previous ocular surgery
- Steroid usage.

### Risk factors for disease progression

- Family history of glaucoma blindness
- Severe visual loss at presentation
- Previous history of high IOPs.

### Medical factors in glaucoma management

- Contra-indications to medications. For example, topical beta-blocker therapy (such as Timolol) is contra-indicated in people with asthma, chronic pulmonary disease, and cardiac disease (heart block and bradycardia)
- Physical difficulty in instillation of eye drops, for example in people with severe hand arthritis, injuries, or poor vision.

**Van Herick's technique, step by step**

The depth of the anterior chamber measured at the temporal limbus is a good indicator for the risk of angle closure. Van Herick's technique involves using a slit lamp to estimate the depth of the anterior chamber at the temporal limbus by comparing it with the peripheral thickness of the cornea at this point.

The technique should be performed in a standardised way so that results can be compared at different points in time or between patients.

**The steps (Figure 1)**

**Figure 2:**
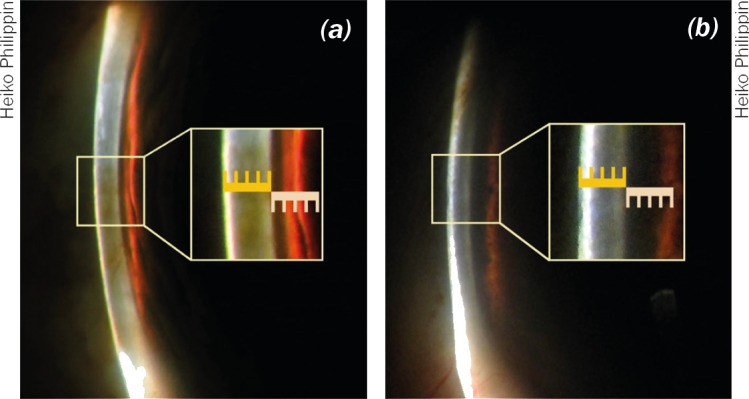
A temporal limbal chamber depth of (a) 25% and (b) 75% of peripheral corneal thickness.

1. Explain to the patient what you are going to do.
2. Dim the lights in the room.
3. Turn the illumination column of the slit lamp to the temporal side, away from the visual axis, by 60**°**. Some slit lamps can lock at this angle (Figure 1).
4. Shine the slit lamp beam from the side at the peripheral part of the cornea and iris (the limbus), where the anterior chamber and iris are just visible. The light must be perpendicular to the temporal limbus, as close as possible to the limbus.
5. View the anterior chamber from the nasal side.
6. Compare the depth of the anterior chamber with the peripheral corneal thickness (Figure 2).

**Figure:**
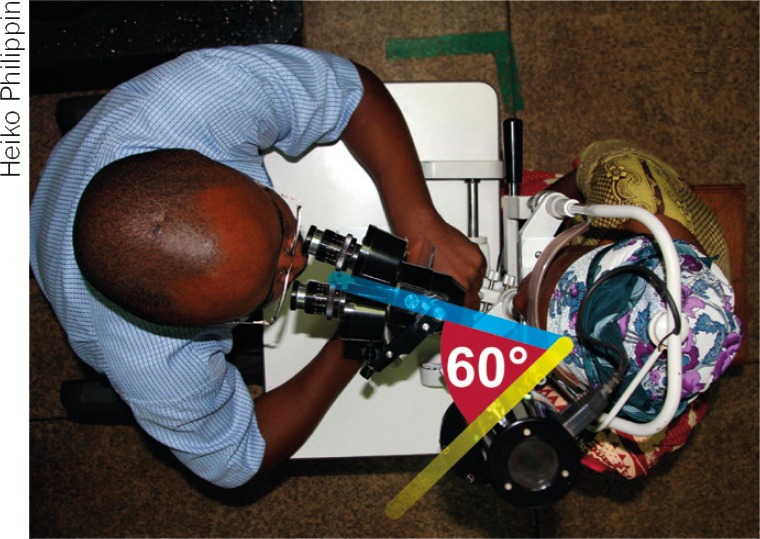
Figure 1

If the depth of the temporal limbal chamber is less than a quarter of the peripheral corneal thickness, then there is a high likelihood (around 84%) that the person has an occludable angle in that eye. If the thickness of the temporal limbal chamber is less than 5% of the depth of the chamber, the likelihood that it is angle-closure glaucoma increases to around 91%.

**Further reading**

1. Foster PJ, Devereux JG, Alsbirk PH, et al: Detection of gonioscopically occludable angles and primary angle closure glaucoma by estimation of limbal chamber depth in Asians: modified grading scheme. Br J Ophthalmol 2000; 84: 186–192.

### Social factors in glaucoma management

- Ability to afford long-term medication
- Access to a pharmacy to obtain repeat drug prescriptions
- Social and family support
- Contact with other health care workers or traditional healers
- Visual requirements for daily activities
- Level of social deprivation
- Geographical isolation with respect to medical services
- Age or life expectancy.

## Visual acuity

Accurate measurement of visual acuity (VA) is critical in glaucoma. Distance VA is normal in most patients with glaucoma unless the disease is advanced. Rule out refractive errors if VA is reduced. Reduced best-corrected visual acuity in mild or moderate glaucoma should alert the clinician to an alternative co-pathology (such as cataract, central retinal vein occlusion, retinal detachment, or diabetic retinopathy). Longitudinal analysis of distance visual acuity over time allows the clinician to detect disease progression, cataract, and other problems.

## Visual fields

Testing visual fields to confrontation with a red target (see page 68) can detect significant visual field defects. Simple measures such as tangent screen testing can be effective. Accurate assessment of visual field defects requires visual field perimetry: manual (Goldmann) or automated (Humphrey) perimetry techniquesgive detailed visual field data. The results of these are dependent on the experience and skill of the person doing the tests, however.

## Slit lamp examination of the anterior segment

A systematic examination of the anterior segment ensures that all important clinical signs are observed.

Gonioscopy is very helpful, however, if a gonioscope is not available, the depth of the limbal anterior chamber can be estimated by Van Herick's test (see the panel opposite). See Table 1 overleaf for a standardised glaucoma assessment tool.

## Gonioscopy

The anterior chamber angle drains most of the aqueous fluid, hence it is essential to assess it in cases of suspected glaucoma. Gonioscopy contributes answers to two questions:

1. What type of glaucoma is it?
2. What is the risk of angle closure?

**Gonioscopy, step by step**

**Figure 3:**
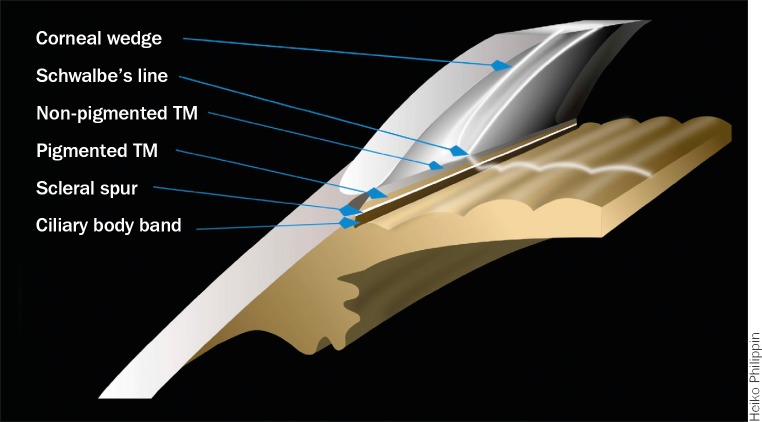
Cross-section of the chamber angle. Abbreviation: TM: trabecular meshwork

There are two types of indirect goniolenses.

The Goldmann lens needs a coupling fluid. When indenting, the patient has to look towards the mirror. It gives clear views of 360° of angle with rotation. The four-mirror goniolens does not require a coupling fluid. Indentation can be performed in primary gaze, but the lens is unstable on the cornea. Corneal folds therefore develop easily and may reduce the clarity of angle structures.

1. Gonioscopy needs to be done in a dark room with a short slit lamp beam. Shining the beam directly into the pupil should be avoided, as this may change the angle configuration, changing a narrow angle to an open configuration.
2. The mirror is placed at 12 o'clock in order to visualise the inferior angle, which is usually more open.
3. Angles are best graded from anterior to posterior. An anterior landmark which serves as a starting point is Schwalbe's line (Figure 3): the end of Descemet's membrane between the corneal endothelium and trabecular meshwork. It can be located with the optical corneal wedge: if a narrow slit beam is tilted showing the cornea in full thickness, the reflections from the anterior and posterior surfaces of the cornea meet at Schwalbe's line.
4. The goniolens is then rotated to view 360**°** of the angle.
5. If the iris has a convex configuration and obscures angle structures then the patient can be asked to look towards the mirror.
6. Indentation is helpful if the angle is narrow or closed. If the angle is closed by adhesions, it will not open on indentation (synechial closure). If the angle is closed only by apposition, it will be forced open and reveal the recess on indentation.

**Figure:**
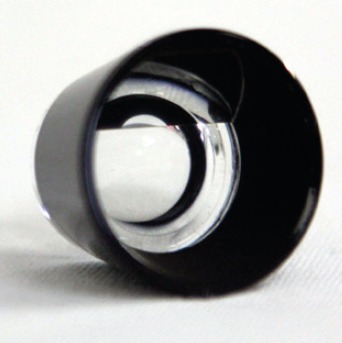

**Figure 4:**
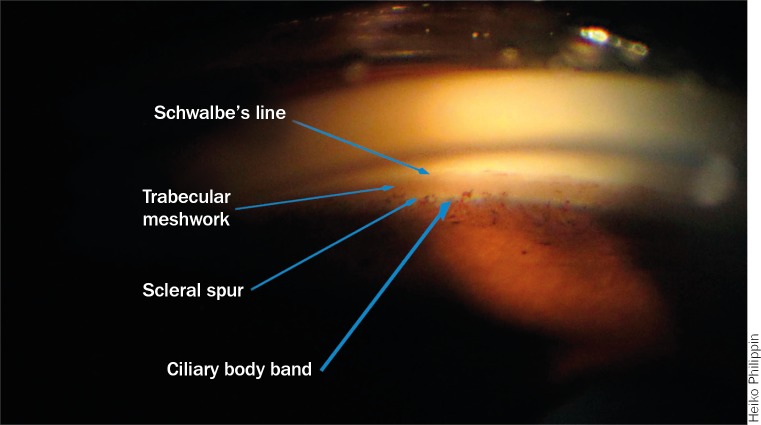
Open chamber angle of an African patient viewed with a gonioscope

**Table 1:** Standardised glaucoma assessment tool. Abbreviations: XFG: pseudoexfoliation glaucoma; PG: pigmentary glaucoma; UG: uveitic glaucoma; LG: lens-induced glaucoma; NG: neovascular glaucoma; TG: traumatic glaucoma; OSD: ocular surface disease

What to examine?			Why?
**Right eye**		**Left eye**	
	**Visual acuity**: Uncorrected Pinhole With correction		If a reduced visual acuity does not improve after refraction, it is commonly due to cataract or very advanced glaucoma. Alternative or co-pathology must be ruled out
	RAPD Anisocoria Pupils		Asymmetric glaucoma, previous trauma or inflammation of the anterior segment causing posterior synechiae.
	Lid margin		Blepharitis, dry eye as part of OSD
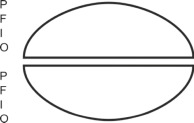	Conjunctiva	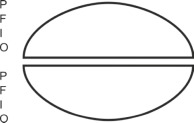	Papillae, Follicles, Inflammation, Oedema, cicatricial disease, vernal keratoconjunctivitis, Bitot's spot, pterygium, conjunctival growth causing ocular surface disease
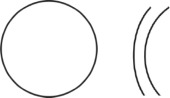	Cornea	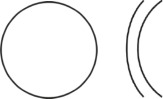	Oedema, scars (TG?), infiltrates, keratic precipitates (UG), central vertically distributed pigment deposits on the endothelium (Krukenberg spindle) (PG)
	Anterior chamber		Depth (van Herick), cells (inflammation) (UG), hyphema, vitreous (TG)
	Iris		Transillumination defects (PG), white flake-like material on the pupillary border, absent pupillary ruff, poor dilatation (XFG), pigment dispersion (PG, XFG), heterochromia, iris nodules, posterior synechiae, atrophy, anisocoria (UG), Neovascularisations of the iris (UG, NG)
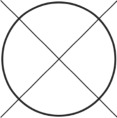	Gonioscopy 1° position	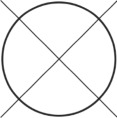	Increased trabecular pigmentation (XFG; PG), debris and peripheral anterior synechiae (UG), fine white protein deposits (LG), trabecular neovascular membrane or fine neovascularisations (NG), angle recession, ghost cells, retained foreign body, cyclodialysis cleft (TG). Angle closure.
	Lens		Luxation (TG), irido-phacodonesis (XFG), large lens, hypermature cataract (LG)
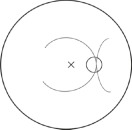	Optic nerve head Macula Periphery	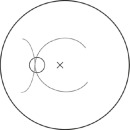	Vertical cup-disc ratio, position of vessels, macular degeneration, diabetic retinopathy, retinal detachment, hypertensive retinopathy

See the panel on page 51 for practical instructions. The morphology of the chamber angle can be classified using several systems. For example, the Shaffer classification grades morphology from 4 to 0, where:

- **Grade 4**: ciliary body band visible, angle wide open
- **Grade 3**: scleral spur can be identified
- **Grade 2**: trabecular meshwork is visible, angle closure is possible but not very likely
- **Grade 1**: only Schwalbe's line visible, high risk for angle closure
- **Grade 0**: angle closure due to iridocorneal contact.

Other important signs are peripheral anterior synechiae, which occur when the peripheral iris adheres to the trabecular meshwork. Peripheral anterior synechiae should not be confused with iris processes, which usually do not cross the scleral spur. Other features to look for are iris or angle neovascularisation, angle recession, cleft, and pigmentation. The angle should be documented in four quadrants. If there is angle closure, additional manoeuvres such as asking the patient to look towards the mirror or intendation will give additional information (e.g. the presence of a plateau iris configuration or synechial closure)

## Tonometry

Accurate IOP measurement together with optic disc assessment is the backbone of diagnosis and management of glaucoma. IOP can be measured with applanation tonometry; this is still the gold standard, but it is difficult to get accurate readings unless the examiner is experienced.^1^ The applanation tonometer also needs to be calibrated regularly. Other instruments for measuring IOP include the Schiotz tonometer, the tonopen, and the non-contact ‘airpuff’ tonometer. Rebound tonometry may be also an alternative if applanation tonometry is not available, and is very useful in children or at mobile clinics.^2^

Normal IOP is below 21 mmHg. However, be aware that patients who return for follow-up visits may remember to use their eye drops just before they come to the clinic, so that theassessed; ir IOP appears to be controlled. This means that the optic disc and visual fields must also be assessed; do not rely on IOP alone.

## Ophthalmoscopy of the optic disc

Glaucomatous changes to the optic nerve head are central to diagnosing glaucoma and its progression. See page 55 for a detailed guide to identifying a glaucomatous optic nerve head.

## Summary

Identifying and documenting the cause of glaucoma, as well as the resulting structural changes and functional loss, are key steps in assessing a patient with glaucoma. They allow the clinician to determine if there are any specific modifiable factors and provide information on the severity of the disease to guide the management decisions.

## References

[1] Whitacre MM, Stein R (1993). Sources of error with use of Goldmann-type tonometers.. Surv Ophthalmol.

[2] Briesen S, Schulze Schwering M, Roberts H, et al (2010). Minimal cross-infection risk through Icare rebound tonometer probes: a useful tool for IOP-screenings in developing countries.. Eye.

